# Breeding Potentials of Bambara Groundnut for Food and Nutrition Security in the Face of Climate Change

**DOI:** 10.3389/fpls.2021.798993

**Published:** 2022-01-05

**Authors:** Oluwaseyi Samuel Olanrewaju, Olaniyi Oyatomi, Olubukola Oluranti Babalola, Michael Abberton

**Affiliations:** ^1^Food Security and Safety Niche Area, Faculty of Natural and Agricultural Sciences, North-West University, Mmabatho, South Africa; ^2^Genetic Resources Center (GRC), International Institute of Tropical Agriculture (IITA), Ibadan, Nigeria

**Keywords:** climate change research, food security, next generation sequencing, Bambara groundnut, underutilized legume, water deficit stress

## Abstract

Constant production of quality food should be a norm in any community, but climate change, increasing population, and unavailability of land for farming affect food production. As a result, food scarcity is affecting some communities, especially in the developing world. Finding a stable solution to this problem is a major cause of concern for researchers. Synergistic application of molecular marker techniques with next generation sequencing (NGS) technologies can unlock the potentials hidden in most crop genomes for improving yield and food availability. Most crops such as Bambara groundnut (BGN), Winged bean, and African yam bean are underutilized. These underutilized crops can compete with the major crops such as cowpea, soybean, maize, and rice, in areas of nutrition, ability to withstand drought stress, economic importance, and food production. One of these underutilized crops, BGN [*Vigna subterranea* (L.), Verdc.], is an indigenous African legume and can survive in tropical climates and marginal soils. In this review, we focus on the roles of BGN and the opportunities it possesses in tackling food insecurity and its benefits to local farmers. We will discuss BGN’s potential impact on global food production and how the advances in NGS technologies can enhance its production.

## Introduction

The evidence of climate change is now overwhelming, with a rise in global temperature predicted by up to 4°C by 2100, with alterations in wind patterns, and precipitation (Thuiller, 2007). According to the United Nations, it involves shifts in weather patterns which affect food production, and rises in sea levels which result in flooding.^1^ Climate change is a danger to the sustainability of food and nutrition. It is one of the most pressing challenges facing food security and nutrition (Abberton et al., 2016). Improving crops for adaptation to climate change effects is essential to avert a looming decline in food production. Hence, the need to develop crops that are resistant to drought stress, salinity stress, higher or colder temperatures, and flooding, which are made more pronounced by climate change.

**FIGURE 1:**
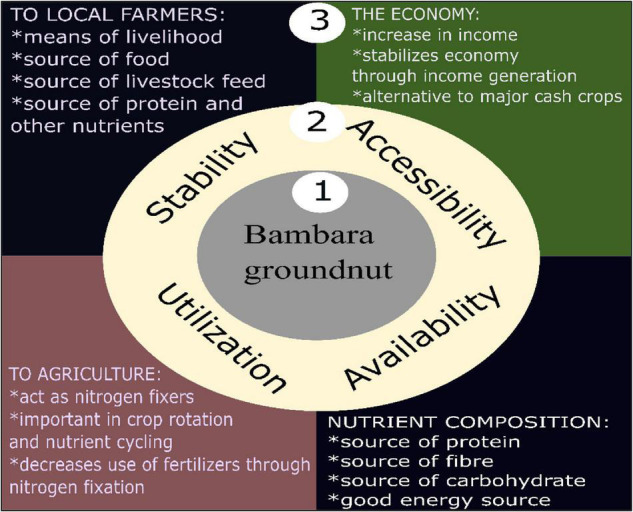
Aligning BGN with the SDG goals. The relationship of Bambara groundnut (1) to the four pillars of food security (2) and the importance of this crop to farmers, agriculture, the economy, and its nutrient composition (3).

The importance of food security in society cannot be over-emphasized but, achieving food security is hampered by climate change impact. According to the World Food Summit (1996), food security exists when sufficient safe and nutritious food is available for people. These foods must also meet the relevant dietary needs to maintain a healthy life. Household food security is applying this concept to the family level, with individuals within households as the focus of concern. However, most households in the developing world do not have daily access to quality food. The FAO, therefore, deemed food security to be achieved based on these four pillars; availability, accessibility, sustainability/stability, and utilization (Figure 1).

One of the highest occurrences of food insecurity and malnutrition is recorded in Africa (Khan et al., 2016). Africa and Asia have been postulated to have the highest population increase by 2050 because most urban growth will be concentrated on these two continents (Huang et al., 2019). This will place severe strain on the available food system; therefore, there is the need for an extensive revolution in agricultural research to mitigate the effect of climate change on food availability in these regions. In a bid to also achieve food availability, some crops which are domesticated in their locality are grown, especially in Africa and some parts of Asia. However, these crops are not widely accepted when compared with their major counterparts. Therefore, they are consumed primarily within their locality. These crops are termed orphan/underutilized/neglected crops grown by subsistence farmers in their local adaptations. They have proven to be vital sources of nutrients and income (Ratnayake et al., 2020), even when faced with the impacts of climate change.

The development of a high-yielding and climate-resilient crop is essential for food security. Although there have been positive improvements from conventional plant breeding, the rate of genetic gain has not been entirely up to expectations (Chen L.Y. et al., 2019; Piunno et al., 2019). Both conventional breeding (such as grafting and crossing techniques) and new technologies [such as genome editing, epigenetics, phenomics, transgenesis, protoplast fusion, and marker-assisted selection (MAS) breeding] can be harnessed to mitigate the impact of climate change on food availability. Furthermore, increased diversity in cultivated plant species will improve the plant germplasm and increase genetic coverage for improvement. Despite the availability of over 31,000 useful plant species, it is surprising that just three crops (rice, wheat, and maize) provide over 50% of plant-derived calories for use (Borrell et al., 2020). However, the custom of planting limited species of a crop is not favorable because these crops can become vulnerable to climate instability. Besides, more crop varieties will allow variability and diversity in improving important adaptive mechanisms and nutritive values, while keeping the present varieties to prevent loss of diversity. Agricultural biotechnological research for the development of more crop varieties has been an important focus area of the Consultative Group on International Agricultural Research (CGIAR) in collaboration with other partners (Hickey et al., 2017). Researchers carried out several collaborative types of research on different staple food crops in various developing countries. These researches, especially those involving the locally adapted underutilized crops, improve the lives of the farmers in these countries.

Due to the predicted increase in the world population, there is the need for an immediate revolution in the agricultural system to satisfy food demand. With underdevelopment and climate change, there is no better solution to combat the looming food scarcity than to look within. Therefore, the improvement of underutilized indigenous crops could proffer a lasting solution to food insecurity in Africa. The best potential underutilized crop must be able to adapt to its environment, have market value, acceptable taste and texture, and must be able to thrive well with less agricultural inputs (Mayes et al., 2011).

Bambara groundnut (BGN) is an underutilized crop with a lot of potentials for achieving food security in sub-Saharan Africa. Africa produces ∼0.3 million tons of BGN annually with an average of 0.85 t/ha. Nigeria, Burkina Faso, and Niger used to be the largest producers in Africa producing 0.1 million, 44,712, and 30,000 tons, respectively (Hillocks et al., 2012), but recently Burkina Faso, Niger, and Cameroon are the largest producers with 74% of the world production (Majola et al., 2021). The lipid content in BGN compares favorably well with that observed in cowpea (1–1.6%), pigeon pea (1.2–1.5%) but lower than groundnut (45.3–47.7%) (Azam-Ali et al., 2001). The predicted yield in Africa is between 300 and 3,000 kg/ha with majority of west African countries predicted to produce in the range of 300–1,000 kg/ha while southern African countries were predicted to produce between 1,000 and 3,000 kg/ha (Azam-Ali et al., 2001). Hence, the highest production of the crop should be from the southern part of the continent. This view is confirmed by Majola et al. (2021), who reported the southern African region as the suitable region in the continent for the production of BGN. Despite its reported qualities, one of the critical challenges facing BGN production and other underutilized legumes is their low yield when compared to the major crops (Sidibé et al., 2020; Temegne et al., 2020). This can result from loss in genetic diversity during domestication, as most of their wild relatives perform better and adapt well over the years (Zhang et al., 2019). But strategic technological advancement in the production of BGN involving genetic analysis can provide essential data for breeding programs that will enhance its potential in improving food and nutritional availability in the presence of low water availability. Furthermore, when other legumes, such as soybean, chickpea, and groundnut, are shifting into the post-genomic era, BGN is just drifting into its genomic era. As a result, available genetic resources for efficient breeding programs are limited.

Hence, this review will be looking at the current knowledge in molecular breeding through next generation sequencing (NGS) technologies, and the progress expected from these technologies on BGN production. We will take a brief look into some of the different NGS technologies that apply to improving crop yield. Each has its specific attributes depending on the research questions to be answered, such as trait mapping and association analysis (Liu and Cheng, 2020), breeding for drought tolerance (Nithya et al., 2020), breeding for salt tolerance (Yu et al., 2020), and genome diversity (Hassani et al., 2020). Finally, we will outline some key points that need to be addressed for BGN to become fully utilized in improving food and nutrition security.

## Bambara Groundnut (*Vigna subterranea* (L.) Verdc.) Provides a Sustainable Alternative to Major Crops

**FIGURE 2:**
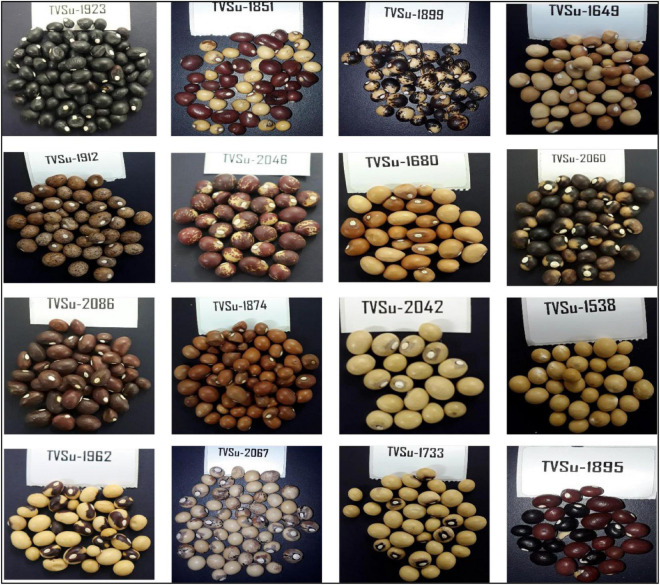
Bambara groundnut seeds showing variability in different accessions through colors, shapes, and eye patterns.

Bambara groundnut is gradually receiving more international research attention. It is considered a complete food containing a high amount of protein and other nutrients (Halimi et al., 2019; Table 1). A pulse with a subterranean fruit set, cultivated by subsistence farmers mostly in the semiarid part of Africa, and an African legume (Mayes et al., 2019) with variations in seed color and morphology: such are the attributes of BGN (Figure 2). The botanical name of the crop is *Vigna subterranea* (L) Verdc, which consists of the wild species type (*V. subterranea* var. *spontanea*) and the cultivated variety (*V. subterranea* var. *subterranea*). Its high carbohydrate amount and considerable protein contents make it regarded as a complete food (Bamshaiye et al., 2011).

**TABLE 1:** Nutritional components of BGN and some underutilized legumes.

Nutrient components	BGN	Cowpea	African yam bean	Winged bean	Mung bean
Moisture (%)	4.30	10.79	8.84	5.55	8.08
Protein (%)	23.59	26.76	21.26	28.52	26.50
Carbohydrate (%)	64.4	50.53	61.92	34.11	56.52
Fat (%)	6.50	0.92	1.76	16.72	1.33
Fiber (%)	5.49	11.03	5.19	5.51	3.67
Ash (%)	4.30	3.12	3.40	4.56	3.91

*Source: BGN (Yusuf et al., 2008; Halimi et al., 2019); Cowpea (Gondwe et al., 2019); African yam bean (Adegboyega et al., 2020); Winged bean (Adegboyega et al., 2019); and Mung bean (Li et al., 2010).*

Bambara groundnut is believed to be the most resilient to drought among grain legumes (Ntundu, 1997; Jørgensen et al., 2010). Wild varieties predominate due to limited research into domesticating new varieties. The major producing/exporting countries are Niger, Ghana, Chad, Nigeria, Mali, Senegal, Côte d’Ivoire, Burkina Faso, and Togo. The International Institute of Tropical Agriculture (IITA), also in Nigeria, holds over 1900 accessions obtained from various countries in their Genetic Resources Center (Paliwal et al., 2020). Knowledge of the genetic variation of BGN accessions will be important for their efficient use in breeding program, studies on the crop’s evolution, and conservation purposes. BGN is characterized by a high degree of variability for various morphological, physiological, and agronomic traits. To analyze the genetic structure of crop germplasm, the estimation of variation within and between populations of its species is important. Genetic diversity on the crops has been studied using various molecular markers (Olukolu et al., 2012; Fatimah and Ardiarini, 2018; Konate et al., 2019), and more researches are still ongoing in this regard. As landraces are not available, BGN varieties must still be developed; thus, their continued improvement and development contributes to the development of diverse, superior varieties. Mutant collections made using the tools of genetic engineering can supply new genetic diversity as well.

Bambara groundnut belong to the genus *Vigna*, subclass *Phaseolinae*, class *Phaseoleae* and family *Leguminosae* (Azam-Ali et al., 2001; Bamshaiye et al., 2011). It was known as *Voandzeia subterranea* for more than a century before it was changed to *V. subterranea* in 1980 (Borget, 1992). A comprehensive botanical study by Maréchal et al. (1978), found striking similarities between BGN and plant species of the genus *Vigna* leading to the studies by Verdcourt who later proposed the name *V. subterranea* (L) Verdc (Goli, 1995).

## Mainstreaming Bambara Groundnut for Ensuring Food and Nutritional Security

Bambara groundnut plays an important role not only in addressing food and nutritional well-being, but also in boosting immunity (Adebowale et al., 2011; Adedayo et al., 2021), livestock feed (Feldman et al., 2019), improving biodiversity, and protecting farmers’ livelihoods. Resistant starch is concentrated in this crop, which encourages the slow and sustained release of glucose into the bloodstream, making a healthy diet (Cummings and Englyst, 1995). These characteristics highlight the significance of BGN being introduced into the mainstream. Prioritizing the development of the best agronomic practices, improving storage and supply chains, and using better methods of delivery for BGN is possible, but it will necessitate developing a roadmap that includes developing beneficial traits, utilizing better agronomic practices, and optimizing supply chains. Identifying suitable BGN species for cultivation and providing training to farmers is required at hotspots where other species are being grown.

When searching for an appropriate variety, location, climatic conditions, soil fertility, and availability of human resources all play a role. If there can be easy access to worldwide germplasm repositories, releasing seed materials for cultivation will be simpler; however, improvements in target location may be required to produce higher agricultural values of the produce. Because of the availability of recent and relevant genetic tools and biotechnological interventions, it would be beneficial to work with modern genetic engineering techniques and biotechnological interventions to aid in the BGN breeding process. Simple crop management techniques, including straightforward irrigation techniques, soil preparation, and crop rotation, help to produce good crops. Using this techniques thus enable higher yields and avoid crop failure (Muthamilarasan et al., 2019). Insect pests that have been observed to be attacking BGN have been reported (Lale and Vidal, 2003; Majola et al., 2021). An understanding of these pests’ biology, including their mode of action, and the various host defenses that organisms use to resist insect pests is critical for the development of insect-tolerant varieties. Mitigating the effects of insect pests can also be helped by optimizing integrated pest management strategies.

Large scale production of seed grains can encounter bottlenecks in post-harvest processing and storage, but streamlined processes and equipment are available to minimize processing and storage loss, as is the case with BGN. Crop-specific technologies are required to develop and implement solutions that are specific to each BGN grain type. Long-term storage of BGN grains necessitates the use of protective measures such as storing BGN grain at temperatures far beyond the optimal range to avoid sprouting or rancidity, and as a result, valuable produce gradually turns rancid. This is attributed to the oxidation of the unsaturated fatty acids (Adeleke et al., 2018). To mitigate global hunger and child malnutrition indices, these storage facilities can be assessed by people during natural disasters or future pandemics. Incorporating an established supply chain helps ensure the agricultural outputs, such as BGN, that benefit farmers and stakeholders who are involved in small scale BGN cultivation can be distributed throughout the supply chain.

## Biotic and Abiotic Factors Affecting Bambara Groundnut Production

**FIGURE 3:**
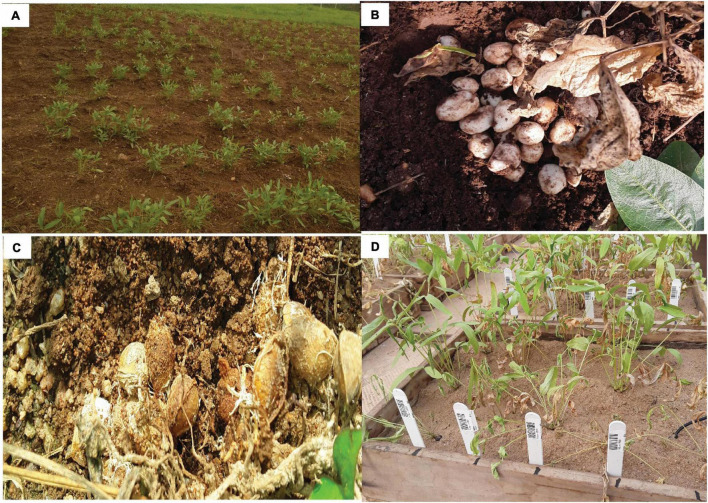
**(A)** Bambara groundnut (BGN) on the field; **(B)** healthy matured BGN pods during harvest; **(C)** infected BGN pods; **(D)** drought stressed BGN plants.

Diseases and pest infestation on the crop are not very pertinent. Therefore, reports have not been elaborate on it. Ability to produce their foods below the soil and the hardness of the seeds aid in their resistance to pests. Notwithstanding, BGN still hosts pathogens and insect pests which cause a significant economic impact through yield loss (Figure 3). Major biotic constraints to BGN production are disease, insects, and viruses. There are diverse stands as regards the crop being affected by pests and diseases. Purseglove (1992) stated that the crop is free from pests and diseases meaning that it cannot be affected by any pest or disease. This notion is partially supported by Gibbon and Pain (1985). However, Gibbon and Pain (1985) also reported the possibility of infestation by leafhoppers (*Hilda patruelis* and *Empoasca facialis*). Tanimu and Aliyu (1990) in their own view observed that the crop is free from other insect pests that affect other legumes such as cowpea and peanut. This limits the use of a pesticide when cultivating the crop. Goli (1995) reported the attack of BGN pods by termites. Immature pods can be damaged by moth beetle (*Piezotraachelus ugandum*), while larvae of *Rivellia* causes damage to the root nodules. Reports of insect infestation on the crop mainly during storage has been reported in the works of Kabir et al. (2017); Nyamador et al. (2017), Mahama et al. (2018), and Moussa et al. (2018).

Moving away from insect pests, Brink et al. (2006) reported that BGN can be infested by fungal pathogens resulting in fungal diseases such as cercospora leaf spot (*Cercospora* spp.), powdery mildew (*Erysiphe polygoni*), and Fusaruim wilt (*Fusarium oxysporum*). Root-knot nematode (*Meloidogyne javanica*) attack the crop in sandy soil (Fourie et al., 2017). In addition, viruses such as cowpea mottle virus (Hema et al., 2014), necrotic mosaic virus (Masindeni, 2006), white clover mosaic virus (Adu-Dapaah et al., 2004) and two potyviruses (Mkandawire, 2007) have been reported to affect the crop. When stored while it is damp, molds are able to grow on the seeds thereby permitting weevils to be able to attack the seed. Bruchids (*Callosobruchus maculatus*) are the most important pest attacking the seeds of the crop during storage (Kosini and Nukenine, 2017).

Aside from pests and diseases, various environmental factors also pose some challenges to the yield of the crop. Factors such as temperature, drought, and salt (Suzuki et al., 2014), affects its growth at different developmental stages (Figure 3). These factors reduce the yield of crops worldwide by as much as 50%. The stresses resulting from the impact of these factors causes morphological, physiological, biochemical, and molecular alterations in the crop which affects its productivity and yield (Bita and Gerats, 2013). In BGN, biotic stress causes yield reduction, but abiotic stress is the most limiting factor causing unstable yield. Important abiotic stresses are temperature, water, soil conditions, and drought stress.

Soil texture and structure that enhance aeration in the soil determine the suitability of soils for BGN production. The seeds of BGN are borne below the soil surface; therefore, the choice of soil type is very important. The crop prefers well-drained, sandy loam soil because they can utilize lighter rain showers to greater advantage than clay soil and the soil cannot damage the seeds (Swanevelder, 1998).

## Recent Progress in Bambara Groundnut Research and Room for Improvement

Crop improvement depends on access to genetic resources. Genetic resources provide parent lines for characterization in breeding programs. Hence, effective use of these lines is of high importance in improving crops. In BGN, available genetic resources have been utilized in various studies which have resulted in the development of molecular markers for trait mapping and possible improvement. However, the lack of reference genome for BGN has hindered the progress of improvement in this regard. Till date, whole genome sequencing generates molecular data that can be used in comparative genomics analysis between underutilized legumes like BGN and their major crop counterparts to identify genes, alleles, and quantitative trait loci (QTL) for yield-determining, agronomic, and climate-resilience traits [reviewed by Khan et al. (2016)]. This techniques was used by Chai et al. (2017) to identify QTL involved in agronomic and drought related traits using an expression marker-based genetic map based on major crop resources developed in soybean. In another study, Ho et al. (2017) carried out a comparative genomics study based on common bean, adzuki bean, and mung bean genomes to identify conserved genes in BGN. The use of molecular markers has resulted in the development of large scale, genome-wide molecular markers, high-density maps, and genomic regions governing key traits (Ahmad et al., 2016).

The primary goal of BGN or any plant breeding program is to alleviate production constraints. Owing to limited genome data, most genetic traits have received less attention in BGN research. It was notable that only the genetic diversity studies from molecular markers have been studied in BGN. No reported genome wide association study on BGN has been reported while very few have identified genes for some traits using comparative genomics studies (Ho et al., 2017). This research should be expanded to aid in the development of genomics-enabled breeding for the improvement of higher yield and higher nutrient-containing varieties. Another intriguing feature of BGN grain is the presence of resistant starch and its hard-to-cook phenomenon (Oyeyinka et al., 2017; Akintayo et al., 2021), which will require further investigation to uncover its genetics and genomics. In comparison, research on nutrient composition (Hlanga et al., 2021), antinutrient composition (Adegboyega et al., 2021), amino acids composition (Oyeyinka et al., 2019), antioxidant composition, responses to environmental stress (Bonthala et al., 2016; Khan et al., 2017), and photoperiodism (Kendabie et al., 2020), has been conducted in BGN. Achievements that further genomic tools such as molecular markers, gene editing, omics will bring to BGN breeding are discussed elsewhere in this study by comparing the impact of these tools on some major crops.

### Key Points to Be Addressed in Bambara Groundnut Improvement Include

#### Tackling Low Yield Syndrome

Underground pods are a big challenge for the yield output in this crop. The seeds must be covered or else they will not mature. This exposes them to waterlogging during heavy rainfall; it can also expose them to too much heat in hot climates. Because of their high level of oil content, excessive heat is not good for the embryo. This situation can be helped through genotype by environment interaction studies. Accessions should be assessed for their performance in various environments and accessions with the best and stable yield at the various environments could be developed for improved varieties (Olanrewaju et al., 2021). Accurate records of exact maturity dates should also be taken so that the pods will not overstay in the soil before they are harvested. Post-harvest procedures such as threshing and storage pose another great challenge to the output of this crop. There is no mechanized way of threshing; when done manually, most of the seeds are destroyed and a large portion of the harvest is lost. Finding better ways to prevent these significant losses will boost total output.

#### Research Funding

Until recently, research funding for underutilized crops have been limited when compared with their counterparts among major crops. Most organizations prefer to support widely known crops. The availability of funds for research on these crops will not only reveal their importance, but will also create an opportunity to generate more varieties from their wild counterparts. Domestication will be made easier as the latest advances in technology will open new frontiers. The role of biotechnology in the preservation of germplasms cannot be over-emphasized. Collections of these germplasms allow the selection of traits for improved breeding (Dawson et al., 2009). Therefore, technologies such as gene editing, genomics, proteomics, transcriptomics, and other omics technologies can all help to improve different varieties of BGN for various useful traits such as drought tolerance, salt tolerance, nutrient composition, and physical traits.

#### Favorable Government Policies

Most countries in the developing world import most of their food. Policies can be set by the government for a gradual reduction in importation. This will lead to the consumption of the local underutilized crops such as BGN until they gradually become fully accepted. An increase in awareness of the benefits of BGN leads to an increase in consumption. Once demand has increased, there will be a definite increase in the cultivation and production of these locally grown crop species. Therefore, there should be a favorable balance between policies governing exports and imports of food crops (Tomlinson, 2013). Supporting exports of the underutilized crops will be a source of revenue for the nation. This will improve the economy and provide another source of income generation.

#### Crop Popularity Issues

Most people are not aware of these underutilized crops because most of them are grown only in the immediate locality of their use. They are not widely available to the larger part of society (Jaenicke, 2013). Awareness should be created for people to know and have access to these crops as supplements to the popularly known counterparts. The various media devices available can be used to create the needed awareness.

#### Unavailability of a Reference Genome

The availability of a complete genetic map of BGN will increase speed breeding in this crop. As at the time of this study, the only established one is a draft genome, which was reported in the study of Chang et al. (2018b). This does not represent a full genome, as coverage is incomplete and the assembly is fragmentary. Complete coverage will give detailed knowledge of the crop which will inform breeders about various traits that can be improved for better production.

#### Awareness of the Right Planting Period

Finally, the BGN needs water for stabilization on the field, after which it needs little or no water. Farmers need to be wary that it does not need much water, especially once it starts podding. Because of this, planting time should be in such a way to conform with the reduction of rainfall, such that when the crop reaches the podding stage, the amount of rain would have been significantly reduced to help in the quick maturation and drying of the seeds.

## Improving Bambara Groundnut Production: A Case for Genotyping by Sequencing

Yield variation can make the effective use of BGN and other underutilized crops complicated. Thanks to advancements in the use of molecular markers in plant breeding, crops are able to produce greater yields (Zhang et al., 2017). With the aid of these markers, target loci will be located and amplified for further studies. Research to develop disease resistance, stress tolerance, and nutrient and water-use efficiency is important in crop breeding (Wani et al., 2016; Khan et al., 2020). Therefore, development of an improved BGN variety is the focus for BGN breeding. The greater the population grows, the greater the demand for food. Genomics-based innovations in NGS have expedited crop research and provided access to the previously closed frontiers of functional genomics, gene discovery, and molecular marker development in non-model plants. Through the construction of linkage maps (Meng et al., 2015), characterization of traits *via* QTL determination leading to MAS was a major achievement, causing a revolution in plant breeding, hence its translation into BGN breeding will be a success for achieving food security. Markers linked to a gene can be developed after the gene of interest has been identified through genomic studies or other applications. Similarly, the parallel sequencing of RNA (RNAseq or transcriptome profiling) is a powerful tool for transcription profiling, providing rapid access to a large collection of expressed sequences (transcriptomes). RNAseq technology has been successfully applied in several organisms, including model and non-model plants (Wang et al., 2017). It can be used as a cost effective source for developing molecular markers such as Simple Sequence Repeats (SSRs) and Small Nucleotide Polymorphisms (SNPs). It is expected that these transcriptome-derived markers will show greater transferability among closely related species than the genomic markers because of their presence in more conserved transcribed regions of the genome. These markers can also be used for comparative mapping and evolutionary studies between BGN and its closely related *Vigna* species. The complete sequencing of BGN genomes with the assistance of NGS technology will be a milestone for BGN biology and provide needed resources for its functional genomics.

A high number of molecular markers is of great importance in crop breeding. In many underutilized crops, however, the availability of sufficient molecular markers is lacking. Going forward, Diversity Array Technology (DArT) has been the “go-to” technology when it comes to developing molecular markers in underutilized crops basically because there is no need for prior sequence information. The combination of DArT with NGS resulting in DArTSeq has been used in many underutilized crops, including BGN (Ho et al., 2017; Redjeki et al., 2020) for SNP discovery.

Going forward, incorporating gene editing into BGN breeding is a possibility that is worth considering. Sequence-specific nucleases which include engineered homing endonucleases or meganucleases, zinc-finger nucleases (ZFNs), transcription activator-like effector nucleases (TALENs) and the CRISPR-Cas system are used in genome editing (Gao, 2021). Protein engineering in ZFNs and TALENs is expensive, time consuming, and difficult, therefore limiting their use. These challenges have made CRISPR to be a more attractive proposition. It is faster, more precise, cheaper, and efficient. It is more versatile and has been employed in many legumes such as *Medicago truncatula* (Meng et al., 2017; Zhang H. et al., 2020), *Lotus japonica*, *Glycine max* (Zhang P. et al., 2020), and *Vigna unguiculata* (Ji et al., 2019). Removing unwanted trait elements for an improved crop is a strategy used in crop genetic improvement. This is achieved by knocking out genes of undesirable traits. Knocking out undesirable traits is the most common application of the CRISPR-Cas system (Chen K. et al., 2019). Traits such as yield, biotic, and abiotic stress resistance have been improved in crops through CRISPR-Cas application (Li R. et al., 2019; Makhotenko et al., 2019; Bouzroud et al., 2020; Zeng et al., 2020; Zheng et al., 2020; Li et al., 2021; Liu et al., 2021).

Yield is a complex trait and is controlled by factors such as grain number, grain size, grain weight, and panicle size; therefore, targeting the genes that regulate these traits through detection and gene-knock-out in BGN will improve its yield. In the study of Liu et al. (2021), for example, grain yield traits in maize were improved by editing CLE genes using the CRISPR-Cas application. Also, Gn1a and GS3 genes, which regulate grain number and grain size, respectively in rice, were targeted for yield improvement in the study of Li et al. (2016) using the CRISPR-Cas system. Therefore, translating such studies into BGN will provide insight into the mechanism of yield development thereby facilitating its molecular breeding for improved yield.

Bambara Groundnut is rich in nutrient compositions. The nutrients in it are sufficient that it is regarded as a complete food (Bamshaiye et al., 2011; Jideani and Diedericks, 2014). However, BGN possesses a considerate amount of antinutritional factors such as phytates, tannins, and saponins. These antinutritional factors affect protein digestibility and bioavailability of other nutrients (Halimi et al., 2019). Therefore, the removal of these antinutritional factors should be a target in BGN breeding. CRISPR-Cas technology can be applied to target genes regulating the production of these antinutritional factors. The application of CRISPR-Cas technology has been shown to improve nutrient components in seeds of soybean (Li C. et al., 2019). Abiotic stress is a complex trait controlled by many genes. CRISPR-Cas has been applied to induce and edit genomes for abiotic stress genes (Shi et al., 2017; Li R. et al., 2019). Gene-directed mutagenesis can be incorporated in BGN breeding to improve its drought tolerance capability.

Genome editing can be used to modify traits BGN to accelerate the process of domestication. However, the lack of a well-annotated genome, lack of genetic transformation methods, and suboptimal tissue regeneration protocols are bottlenecks underlying the application of gene editing tools on BGN and other orphan crops.

## Prospects for Genomic Markers in Bambara Groundnut Breeding

A vast array of molecular markers has been developed in plant breeding for various purposes. The first set of markers include the Restriction Fragment Length Polymorphisms (RFLPs) which was developed in the early 1980s (Hu et al., 2020), Random Amplified Polymorphic DNA (RAPD) (Yan-Qiong et al., 2019), and Amplified Fragment Length Polymorphism (AFLP) (Yan-Qiong et al., 2019). DArT, SNP, and SSR, which have been regarded as second generation markers, have all been developed and implemented in modern plant breeding technology. Application of molecular markers are pronounced in MAS and genetic plant breeding.

Analysis of genetic diversity in crop species is based on the different phenotypic markers which are affected by the environment making it less sufficient for proper germplasm characterization. Molecular markers, on the other hand, occur at high frequencies and allow for in-depth genetic characterization. Hence combining molecular markers with phenotypic markers allow for a comprehensive characterization of germplasm collections. Even though BGN has a large genetic diversity, there are few molecular markers and genomic resources available. Meanwhile, DNA markers have been used to successfully to analyze genetic diversity in BGN germplasm collections from different parts of the world (Amadou et al., 2001; Ntundu et al., 2004; Somta et al., 2011; Olukolu et al., 2012; Fatimah and Ardiarini, 2018; Konate et al., 2019). The recently developed SNP markers enhance the effectiveness of genotyping because of their high density (Savadi et al., 2020). Therefore, this will aid large scale BGN germplasm characterization, high resolution genetic and QTL mapping.

The world’s largest collection of BGN is held in GRC-IITA and various evaluation studies are being carried out (Paliwal et al., 2020). Large germplasm collection cannot be effectively characterized; therefore, a subset termed “core collections” is taken from the larger collection. The core collection can then be improved for future breeding. MAS through QTL mapping has been used to identify genes controlling important economic traits of interest in BGN and other crops. Genome-wide association studies (GWAS) is more robust and accurate in detecting QTL controlling important traits. In recent years, it has emerged as the best choice for QTL mapping in plant species (Sonah et al., 2015; Tao et al., 2020; Zhang X. et al., 2020). So far, there are no GWAS studies on BGN. However, GWAS can aid accurate detection of QTLs for important traits and their application in molecular breeding of BGN for developing improved climate-resilient varieties.

Furthermore, strategies for the development of molecular markers and their applications are based on polymorphism among individual organism genomes, hence, the nature and type of polymorphism are important. There has been full sequencing of many plant genomes such as Arabidopsis (Michael et al., 2018), rice (Du et al., 2017), soybean (Rehman et al., 2018), barley (Beier et al., 2017), maize (Jiao et al., 2017), and cowpea (Spriggs et al., 2018) but no full sequencing of BGN genome has been reported yet. The use of molecular markers for genetic diversity studies have been reported in BGN (Massawe et al., 2002, 2003; Ntundu et al., 2004; Somta et al., 2011; Olukolu et al., 2012; Siise and Massawe, 2013; Molosiwa et al., 2015; Fatimah and Ardiarini, 2018; Konate et al., 2019; Alhamdi et al., 2020).

In addition, complete pseudochromes of the BGN genome are not yet available because the sequences have been done using high-density Illumina short read data. However, plans are being made to use long read sequence data (Gregory et al., 2019). When compared to cowpea, the BGN total genome size is smaller (Lonardi et al., 2019). While Chang et al. (2018a) reported that it has higher number of protein-coding genes (31,707) than mung bean (22,427) but lower than adzuki bean (34,183), the percentage of BGN that has been functionally annotated is reportedly 98%.

## Bambara Groundnut Production: Drought and Water Stress as a Case Study

Agricultural drought (AD) refers to the reduction of the water level in the soil to a point below the required amount needed by plants. AD causes an increase in soil acidity as well as affecting plant nutrient uptake. Plants need water to transport nutrients from the soil through the roots to other parts where they are needed. This decreased water level makes AD a limiting factor in the improvement of plant production. The ability of BGN to survive where other plants cannot do so has made it important for small scale farmers, especially in the developing world. The farmers benefit from the low maintenance attributes. Both physical and molecular traits have been identified and reviewed for drought, and water stress tolerance in plants (Sahebi et al., 2018), and research focusing on these traits is the right direction for improving BGN production. The response of plants to drought is determined by the duration of water stress, the plant’s developmental stage at the time of stress occurrence, and its genetic and phenotypic make-up.

Though BGN is considered a drought-tolerant crop, limited rainfall can still hamper its productivity. Drought reduces crops’ resistance to pests and diseases as well as nutrient uptake. Due to inter- and intra-accession variations in genetic diversity, there will be variations in the responses of BGN accessions to drought. However, little information is available to understand the genetic basis of the drought tolerance mechanism of BGN.

### Identifying the Genetic Bases for Drought Tolerance in Bambara Groundnut

**FIGURE 4:**
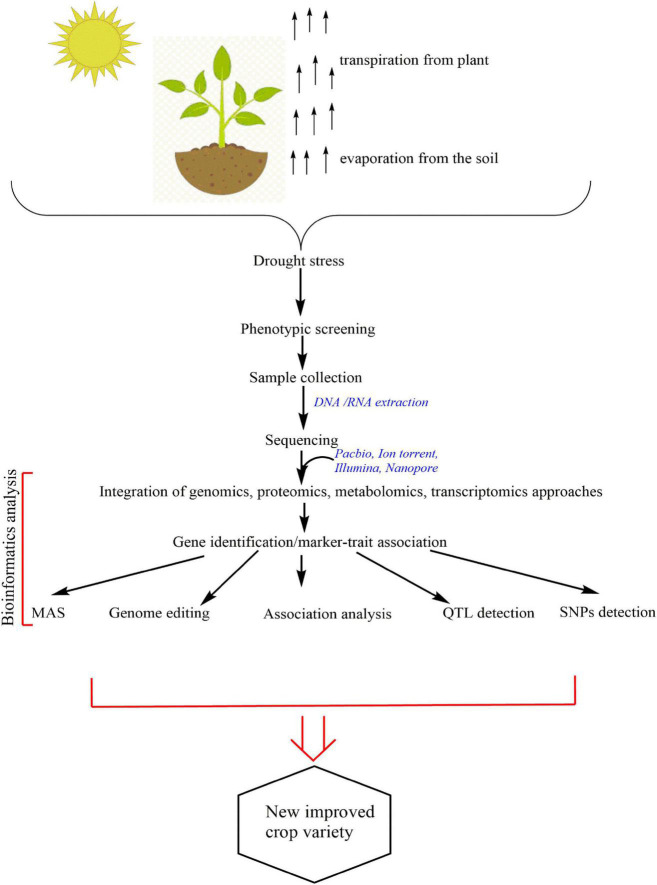
Schematic representation of drought assessment.

A schematic representation for studying drought tolerance in BGN is shown in Figure 4.

The application of these techniques to BGN will help breeders in the selection for highly drought-tolerant landraces; it will help in gene location and editing to create new, improved varieties. Extensive data sets generated from sequences are analyzed using the proper bioinformatics applications and pipelines. Whole genome association studies and genome sequences for functional genomics analysis, co-expression analysis, and QTL mapping all require an advanced level of knowledge on bioinformatics because of the large data sets. System biology approaches work in synergy for a better understanding of the morphology, physiology, biochemistry, genetics, and phenotypic traits in plants (Mohanta et al., 2017) as well as their responses to various environmental challenges.

In addition, drought-tolerant genes in BGN can be identified through comparative studies with the drought-tolerant genes in other crops, and secondly through differential expressions of mRNAs in drought-stressed and non-stressed conditions. The second approach was used to identify genes expressed in stressed and non-stressed plants in BGN (Khan et al., 2017). In their study, Khan et al. (2017) identified PRR7, ATAUX2-11, CONSTANS-like 1, MYB60, AGL-83, and Zinc-finger protein genes, and concluded that these genes could serve as a basis for drought stress study in BGN. Over-expression or suppression of these genes will aid in deciphering their roles in BGN drought tolerance. Under stress conditions, plant secrete solutes like proline, polyols, abscisic acids, jasmonic acids, etc. (Ma et al., 2020). These solutes can serve as markers in BGN stress response studies.

Genome-wide association studies can be another useful technique in identifying the molecular basis of drought tolerance in BGN. Linking phenotypes to genotypes is important in deciphering the genetic basis of any trait. Drought tolerance is controlled by many traits (Parvathi et al., 2020), therefore, it will not be straight forward in regulating loci for all the associated traits. However, GWAS can be used to identify the regions of the chromosome where the loci controlling these traits are present. Identification of these regions will be a starting point for identification of drought tolerance mechanisms in less researched crops with no reference genome, like BGN.

## Conclusion and Recommendations

Modern techniques of improvement are not fully employed in orphan crops. Their crop breeding depends mostly on conventional methods such as selection and hybridization. However, limited numbers of breeders implement modern techniques such as marker-assisted breeding and transgenics. Genomic information such as the whole genome sequence has not gathered pace for any orphan crop. To feed the ever-increasing population in Africa, an agricultural revolution is needed to boost the productivity of orphan crops using modern technologies that have proven to be effective for the major crops of the world.

The application of NGS technology is greatly increasing our knowledge of plant genomics. This has been established in some plants where functional genomics would never have been a reality with earlier sequencing technologies. NGS will enable the quest for higher quality BGN reference genomes and resequencing of many cultivated species genotypes and wild relative accessions soon. With the availability of these rich genetic resources and high-quality genotyping platforms, the functional genomics studies of BGN are entering a new era.

A comprehensive survey of genetic diversity in BGN landraces and wild relatives will deepen our understanding of the genetic basis underlying the domestication and evolution of this orphan crop. Using the NGS technology and informatics combined with information on genetic variation will provide the push needed for BGN to catch up with other crops in studies on functional genomics. The time for genomics-assisted BGN breeding is finally here.

## Author Contributions

OSO researched the data, wrote, and edited the manuscript. OO, OOB, and MA reviewed and supervised the writing of the manuscript. All authors contributed to the article and approved the submitted version.

## Conflict of Interest

The authors declare that the research was conducted in the absence of any commercial or financial relationships that could be construed as a potential conflict of interest.

## Publisher’s Note

All claims expressed in this article are solely those of the authors and do not necessarily represent those of their affiliated organizations, or those of the publisher, the editors and the reviewers. Any product that may be evaluated in this article, or claim that may be made by its manufacturer, is not guaranteed or endorsed by the publisher.

## Footnotes

1
https://www.un.org/en/climatechange/what-is-climate-change
